# Factors associated with HIV testing among youth in Tanzania based on the 2016–2017 Tanzania HIV Impact Survey (THIS)

**DOI:** 10.1371/journal.pgph.0000536

**Published:** 2022-11-11

**Authors:** Yan Wang, Janni J. Kinsler, Sylvia Kiwuwa-Muyingo

**Affiliations:** 1 Division of Infectious Diseases, David Geffen School of Medicine, University of California, Los Angeles (UCLA), Los Angeles, California, United States of America; 2 Section of Pediatric Dentistry, School of Dentistry, University of California, Los Angeles (UCLA), Los Angeles, California, United States of America; 3 Data, Measurement and Evaluation (DME) Unit, African Population and Health Research Center (APHRC), Nairobi, Kenya; Stellenbosch University, SOUTH AFRICA

## Abstract

HIV testing continues to be a challenge among the young population in Tanzania. As of 2017, only 30% of 15–19-year-olds reported getting tested and receiving their results. This study will examine the demographic and socio-behavioral characteristics associated with HIV testing among adolescents and young adults in Tanzania. Interview data from the 2016–2017 Tanzania HIV Impact Survey (THIS) were analyzed on 10,128 adolescents and young adults 15–24 years of age, representing 10.5 million youth in Tanzania. Weighted logistic regression was used to model the relationship of HIV testing with demographic and socio-behavioral characteristics. Half (50%) of respondents reported ever having been tested for HIV. HIV testing was significantly lower among males compared with females (AOR = 0.5;95% confidence interval [CI] = 0.5–0.6; p<0.001), 15–19 year olds compared with 20–24 year olds (AOR = 0.4;95% CI = 0.4–0.5; p<0.001), no education compared with secondary or post-secondary education (AOR = 0.4;95% CI = 0.3–0.6; p<0.001), rural residents compared with urban residents (AOR = 0.7;95% CI = 0.6–0.9; p<0.001) and those who don’t use condoms during sexual intercourse compared with those who do (AOR = 0.6;95% CI = 0.5–0.8; p<0.001). Among HIV-infected youth, younger age group, rural residents, education less than primary, single, high income, and sex workers were significantly associated with never testing for HIV. This study highlights the majority of characteristics affecting HIV testing among young people in Tanzania have not changed over the years, thus it is necessary to re-examine the current approaches to HIV testing. The COVID-19 pandemic will add to this challenge as it collides with the ongoing HIV epidemic and competes for needed medical supplies and health care provider resources. In light of this current situation, intensified and targeted HIV testing programs for at risk young populations in Tanzania should be prioritized.

## Introduction

Worldwide, sub-Saharan Africa continues to have the highest rates of human immunodeficiency virus (HIV), and acquired immunodeficiency syndrome (AIDS) is the leading cause of death among adolescents (aged 10–24) [1, 2]. In Tanzania, data show overall HIV incidence and prevalence rates among adults 15–64 years of age have declined over the past 20 years. Incidence rates have fallen from 0.64% in 2000 to 0.27% in 2017 and prevalence rates have fallen from 7.0% in 2004 to 5.5% in 2017 [3, 4]. However, there are pronounced disparities in rates by gender and age [5, 6]. Tanzania’s HIV epidemic is concentrated among young people, especially girls and young women. In 2018, approximately 24,000 young people between the ages of 15 and 24 were diagnosed with HIV; roughly two thirds of whom were women [7]. The HIV incidence rate among female adolescents 15–19 years of age is significantly higher than male adolescents (0.22% vs. 0.00%) [4]. Prevalence rates for both adolescents and young adults are significantly higher among females than males (1.0% vs. 0.4% for 15–19 year olds and 3.4% vs. 0.9% for 20–24 year olds) [3, 4].

Studies show adolescents and young adults 15–24 years of age are more likely to engage in risky sexual behavior than older adults and have less frequent contact with the healthcare system, thus making HIV testing a challenge for this target population in sub-Saharan Africa [8]. As of 2017, only 30% of 15–19 year olds in Tanzania reported getting tested and receiving their results, while 72% of 20–24 year olds reported getting tested and receiving their results [4]. The Joint United Nations Programme on HIV and AIDS (UNAIDS) 2030 fast-track targets are: 1) 95% of all people living with HIV will know their HIV status; 2) 95% of all people diagnosed with HIV infection will receive sustained antiretroviral therapy; and 3) 95% of all people receiving antiretroviral therapy will have viral suppression. Meeting these targets will be more challenging now than ever given the COVID-19 pandemic that has affected the world. The pandemic has resulted in a disruption of HIV prevention, testing and treatment efforts in many parts of the world, including Tanzania [9]. UNAIDS and the World Health Organization (WHO) have estimated the number of AIDS-related deaths in Sub-Saharan Africa could double if access to healthcare for people living with HIV is interrupted during the COVID-19 pandemic. While interruption to the supply of antiretroviral drugs would have the largest impact of any potential disruptions, the suspension of HIV testing would also have significant impact on the population [10, 11]. Thus, developing novel and creative methods for making HIV testing available and accessible is crucial. Lack of testing and knowledge of one’s HIV status will result in delays in accessing treatment and care and increase the risk of transmitting HIV to their partners, thus increasing HIV incidence and prevalence rates.

In order to help increase HIV testing rates among adolescents and young adults in Tanzania, it is important to understand the factors influencing HIV testing behavior. While structural factors (such as provider capacity, adolescent-friendly confidentiality and privacy protocols, and logistical barriers to HIV testing facilities) affect access to and utilization of HIV testing services [3, 12], demographic and socio-behavioral characteristics play an important role in HIV testing behavior as well. Previous studies conducted among 15–24-year-olds in Tanzania based on the Tanzania HIV/AIDS and Malaria Indicator Surveys (THMIS) between 2003 and 2012 have identified several characteristics associated with a higher likelihood of getting tested for HIV. These characteristics included being female, having a primary or secondary education, older age (20–24 years), having ever been married, living in urban areas, having at least one sexual partner during lifetime and receiving antenatal care [5, 13, 14]. A school-based study among 13–24 year olds showed HIV testing was significantly associated with being female, 18 years of age and older, having an advanced secondary level of education, attending a private school and belonging to a non-Christian religion [15]. Another study among adolescent and young adult females in sub-Saharan Africa (which included Tanzania) found that HIV knowledge, talking about HIV with parents or guardians and having ever been pregnant were associated with an increased likelihood of HIV testing [16].

There is currently a lack of published research on the demographic and socio-behavioral characteristics associated with HIV testing among adolescents and young adults aged 15–24 in Tanzania. This information is necessary to develop targeted approaches to increasing HIV testing among the young population in Tanzania who are disproportionately impacted by HIV. To help address this gap, this study used national data from the Tanzania HIV Impact Survey 2016–2017 (THIS) in which 10% of households had at least one member living with HIV [4]. This study will also discuss the implications of our findings to HIV testing during COVID-19.

## Materials and methods

### Data source and collection

THIS is a national household-based HIV survey that aims to assess the prevalence of important HIV health indicators and the burden of HIV disease [4]. It is part of the Population-based HIV Impact Assessment (PHIA) being conducted longitudinally in 14 countries. THIS Data for the current study was collected between November 2016 and August 2017 in approximately 5,200 households using mobile tablet computers. Adults over 15 years of age completed the adult questionnaire. Adults 18 years of age and older provided verbal informed consent in Kiswahili or English on their own. For youth aged 10 to 17, verbal permission in Kiswahili or English was required from both the youths and their parents to participate in the survey. Electronic informed consent forms were administered using a tablet computer. THIS protocol was reviewed and approved by the institutional review boards of CDC, Columbia University, Westat, and the National Institute for Medical Research and Zanzibar Medical Research and Ethics Committee before interviews were conducted. Detailed consent procedure and protocols were included in the protocol of THIS documents.

### Survey design and sampling weight

THIS is a stratified multistage probability sample design, with strata based on regions and rural status, primary sampling units based on areas within strata, second-stage sampling units based on households within areas, and then participants within households. All eligible adults 15 years of age and older were included in the sample. Details regarding the weighting procedures can be found in the 2016–2017 THIS technical report [4]. There was the potential for nonresponse at different stages during data collection. The non-coverage might have occurred because the list of households was not complete. The overall household response rate was 94.8%. The individual interview response rate was 88.5% among males and 93.2% among females [4]. The final weighting process was adjusted to take into account the nonresponse rate in sampling areas.

### Measures

The outcome variable was whether participants had ever tested for HIV. It was defined as a binary variable (yes/no). In this paper, we focused on HIV testing among adolescents and young adults aged 15 to 24 (about 35% of the 2016–2017 THIS). We included demographic variables, including gender (male/female), residence (urban vs. rural), education (none, primary or less, secondary or more), marital status (never married, married/living together, divorced/separated), and income (low, middle, high). Socio-behavioral variables included age at first sexual intercourse (≤14/≥15), number of sexual partners in past 12 months (0, 1, 2+), condom use during sexual intercourse (yes/no), bought or sold sex for money during the past 12 months (yes/no), sexually transmitted disease (STD) in the past 12 months (yes/no to any of the four type of symptoms asked separately to males and females, such as an abnormal discharge, pelvic pain, and had an ulcer or sore on or near vagina to females, an abnormal discharge from penis and had an ulcer or sore on or near penis to males), alcohol use (never, occasional drinking, heavy drinking) and injection drug use (yes/no).

Respondents were also asked the reasons they did not get tested for HIV. Responses included the following: Don’t know where to test, test costs too much, transport costs too much, too far away, afraid others will know about HIV test results, don’t need to test/low risk, did not receive permission from spouse/family, afraid spouse/family will know results, don’t want to know I have HIV, cannot get treatment for HIV, test kits not available for religious reasons, don’t know, and refused to answer. More than one response could be checked.

### Data analysis

The THIS dataset was requested through the PHIA website under Data Use Agreement. We reported the characteristics of the sample and its representative population. We conducted weighted bivariate and multivariate analyses to determine the covariates that were associated with HIV testing. In the bivariate analysis, we used Chi-square tests to examine the association of HIV testing with demographic and socio-behavioral categorical variables. We then used weighted logistic regression to model the relationship of HIV testing with demographic and socio-behavioral variables. We also conducted a nested analysis to examine the relationship between demographic and socio-behavioral characteristics and HIV testing among HIV-infected adolescents and young adults using weighted logistic regression. The HIV serostatus was determined by a pre-specified HIV testing algorithms that generally included a combination of home-based rapid HIV test and confirmatory laboratory-based testing. All analyses were conducted using SAS 9.4. Additionally, all analyses in this paper were based on weighted results that could be generalized to all of the adolescent and young adult population in Tanzania.

## Results

### Sample characteristics

The final sample included 10,128 sampled observations, representing 10,471,495 adolescents and youth aged 15 to 24 in Tanzania. The unweighted and weighted frequencies for demographic and socio-behavioral characteristics of adolescents and young adults in Tanzania are presented in Table 1. Study participants were equally female (51%) and male (49%). Over half were between the ages of 15–19 (54%), had a primary school education (54%) and were either low or middle income (58%). Approximately three quarters had never been married (70%). Approximately two thirds lived in rural areas (62%).

**Table 1:** Demographic and socio-behavioral characteristics of adolescent and young adults aged 15–25 in Tanzania: Tanzania HIV Impact Survey (THIS) 2016–2017 (N = 10.5 million).

Variables	Unweighted n	Weighted N (%)
Gender		
Female	5,730	5,296,115 (51%)
Male	4,398	5,175,380 (49%)
Age		
15–19	5,314	5,643,926 (54%)
20–24	4,814	4,827,569 (46%)
Education		
No education	897	751,614 (7%)
Primary	5,577	5,645,962 (54%)
Secondary and above	3,654	4,073,919 (39%)
Marital status		
Never married	6,524	7,329,841 (70%)
Married/living together	3,152	2,757,809 (26%)
Separated/divorced	452	383,845 (4%)
Income		
Low	4,153	3,902,908 (37%)
Middle	2,228	2,228,287 (21%)
High	3,747	4,340,299 (41%)
Residence		
Urban	3,506	4,007,443 (38%)
Rural	6,622	6,464,051 (62%)
Age had sex for the first time		
Never had sex	3,053	3,387,149 (32%)
< = 14 years	1,176	1,212,585 (12%)
>15	5,899	5,871,761 (56%)
Number of partners past 12 months		
0	4,051	4,424,080 (42%)
1	4,540	4,358,006 (42%)
2+	1,537	1,689,409 (16%)
Use condoms during sexual intercourse		
No	7,571	7,835,362 (75%)
Yes	2,557	2,636,132 (25%)
Buy or sell sex in past 12 months		
No sexual behavior	3,053	3,387,149 (32%)
Yes	739	749,231 (7%)
No	6,336	6,335,115 (60%)
STD past 12 months		
No	5,986	5,993,686 (85%)
Yes	1,086	1,087,402 (15%)
Alcohol use		
Never drink	9,146	9,488,204 (91%)
Occasional drink	785	781,806 (7%)
Heavy drink	197	201,484 (2%)
Ever injected drugs		
Yes	25	27,489 (<1%)
No	10,061	10,401,154 (>99%)

M = Million

Over half (56%) reported being older than 15 years of age when they had sexual intercourse for the first time. Less than half (41%) reported one sexual partner in the past 12 months and 16% report two or more sexual partners; 75% reported not using condoms when they had sexual intercourse. A small percentage of respondents reported buying or selling sex for money in the past 12 months (7%), heavy drinking of alcohol (2%) and injection drug use (<1%). Almost one fifth (15%) reported an STD in the past 12 months.

### Factors associated with having ever been tested for HIV

Table 2 presents the bivariate results with weighted frequencies for differences in lifetime HIV testing by demographic and socio-behavioral characteristics. Overall, 50% of respondents reported ever having been tested for HIV. Compared with those who have never been tested for HIV, those who have been tested for HIV were more likely to be females compared with males (31% vs. 20%; p<0.001), 20–24 years old compared with 15–19 year olds (34% vs. 17%; p<0.001), those who were never married or married/living together compared those who were divorced /separated (25% and 22% vs. 3%; p<0.001), those with high income compared with those with middle or low income (23% vs. 10% and 17%; p<0.001) and rural residents compared with urban residents (29% vs. 22%; p<0.001).

**Table 2:** Bivariate analysis of ever having tested for HIV among adolescent and young adults aged 15–24 in Tanzania by demographic and socio-behavioral characteristics (N = 10.5 million).

Variables	Ever had HIV test	Never had HIV test
	Weighted N (%)	Weighted N (%)
	5,186,057 (50%)	5,285,437 (50%)
Gender^‡^		
Female	3,239,814 (31%)	2,056,301 (20%)
Male	2,045,624 (20%)	3,129,756 (30%)
Age^‡^		
15–19	1,764,246 (17%)	3,879,679 (37%)
20–24	3,521,191 (34%)	1,306,378 (12%)
Education		
No education	385,792 (4%)	365,821 (3%)
Primary	2,775,027 (27%)	2,870,934 (27%)
Secondary and above	2,124,618 (20%)	1,949,301 (19%)
Marital status^‡^		
Never married	2,646,073 (25%)	4,683,768 (45%)
Married/living together	2,326,968 (22%)	430,841 (4%)
Separated/divorced	312,397 (3%)	71,448 (<1%)
Income^‡^		
Low	1,804,083 (17%)	2,098,826 (20%)
Middle	1,096,635 (10%)	1,131,652 (11%)
High	2,384,720 (23%)	1,955,579 (19%)
Residence^‡^		
Urban	2,269,533 (22%)	1,737,911 (17%)
Rural	3,015,905 (29%)	3,448,147 (33%)
Age had sex for the first time^‡^		
Never had sex	655,407 (6%)	2,731,742 (26%)
< = 14 years	622,624 (6%)	589,961 (6%)
>15	4,007,406 (38%)	1,864,354 (18%)
Number of partners past 12 months^‡^		
0	1,257,632 (12%)	3,166,448 (30%)
1	3,077,410 (29%)	1,280,596 (12%)
2+	950,396 (9%)	739,013 (7%)
Use condoms during sexual intercourse^‡^		
No	3,532,675 (34%)	4,302,688 (41%)
Yes	1,752,763 (17%)	883,369 (8%)
Buy or sell sex in past 12 months^‡^		
No sexual behavior	655,407 (6%)	2,731,742 (26%)
Yes	398,057 (4%)	351,174 (3%)
No	4,231,974 (40%)	2,103,142 (20%)
STD past 12 months		
No	3,896,993 (55%)	2,096,693 (30%)
Yes	731,539 (10%)	355,863 (5%)
Alcohol use^‡^		
Never drink	4,647,821 (44%)	4,840,384 (46%)
Occasional drink	496,238 (5%)	285,568 (3%)
Heavy drink	141,379 (1%)	60,105 (<1%)
Ever injected drugs*		
Yes	7,690 (<1%)	19,799 (<1%)
No	5,254,102 (50%)	5,147,052 (49%)

* P<0.05

† P<0.01

‡ P<0.001

For the socio-behavioral characteristics, those who were significantly more likely to be tested for HIV were participants who reported being older than 15 years of age when they had sexual intercourse for the first time compared with those who were less than 15 years of age (38% vs. 6%; p<0.001), those who had 1 partner over the past 12 months compared to those who had zero or 2+ partners (29% vs. 12% and 9%; p<0.001), those who do not use condoms during sexual intercourse compared with those who do (34% vs. 17%; p<0.001), those who did not buy or sell sex in the past 12 months compared with those who did (40% vs. 4%; p<0.001), those who never drink compared with those who drink occasionally or are heavy drinkers (44% vs. 5% and 1%; p<0.001) and those who have never injected drugs compared with those who have (50% vs. <1%; p<0.05).

Table 3 presents the weighted adjusted odds ratios (AOR) for demographic and socio-behavioral characteristics associated with having ever been tested for HIV. Ever having tested for HIV was significantly lower among males compared with females (AOR = 0.5; 95% confidence interval [CI] = 0.5–0.6; p<0.001), 15–19 year olds compared with 20–24 year olds (AOR = 0.4; 95% CI = 0.4–0.5; p<0.001), no education or primary school education compared with secondary or post-secondary education (AOR = 0.4; 95% CI = 0.3–0.6; p<0.001 and AOR = 0.6; 95% CI = 0.5–0.7; p<0.001), never being married compared with those who are married or living together (AOR = 0.2; 95% CI = 0.2–0.2; p<0.001) and rural residents compared with urban residents (AOR = 0.7; 95% CI = 0.6–0.9; p<0.001).

**Table 3:** Adjusted odds ratio (AOR) for demographic and socio-behavioral characteristics associated with ever having tested for HIV among adolescents and young adults aged 15–24 in Tanzania (N = 10.5 million).

Variables	AOR (95% CI)
Gender	
Female	Reference
Male^‡^	0.52 (0.46, 0.6)
Age	
15–19^‡^	0.41 (0.35, 0.47)
20–24	Reference
Education	
No education^‡^	0.44 (0.33, 0.59)
Primary^‡^	0.56 (0.48, 0.66)
Secondary and above	Reference
Marital status	
Never married^‡^	0.19 (0.16, 0.23)
Married/living together	Reference
Separated/divorced	0.77 (0.54, 1.11)
Income	
Low	0.81 (0.65, 1.01)
Middle	1 (0.83, 1.21)
High	Reference
Residence	
Urban	Reference
Rural^‡^	0.73 (0.6, 0.89)
Age had sex for the first time	
Never had sex	Reference
< = 14 years^‡^	2.12 (1.55, 2.88)
>15^‡^	2.8 (2.18, 3.58)
Number of partners past 12 months	
0	Reference
1	1.23 (0.99, 1.53)
2+	0.92 (0.71, 1.2)
Use condoms during sexual intercourse	
No^‡^	0.65 (0.54, 0.78)
Yes	Reference
Buy or sell sex in past 12 months	
Yes	0.85 (0.67, 1.07)
No	Reference
Alcohol use	
Never drink	Reference
Occasional drink*	1.25 (1.01, 1.54)
Heavy drink	1.5 (0.88, 2.55)

* P<0.05

† P<0.01

‡ P<0.001

For the socio-behavioral characteristics, ever having been tested was significantly lower among those who don’t use condoms during sexual intercourse compared with those who do (AOR = 0.6; 95% CI = 0.5–0.8; p<0.001) and significantly higher among those who were less than 14 years of age or greater than 15 years of age when they had sexual intercourse for the first time compared with those who have never had sexual intercourse (AOR = 2.1; 95% CI = 16–2.9; p<0.001 and AOR = 2.8; 95% CI = 2.2–3.6; p<0.001) and those who are occasional drinkers compared with those who never drink (AOR = 1.2; 95% CI = 1.0–1.5; p<0.05).

### Reasons for not getting tested for HIV

The most common reasons given for not getting tested included: Don’t need to test/low risk (9.8%), don’t know where to test (9.4%), too far away (6.9%), don’t want to know I have HIV (4%), afraid others will know about HIV test results (3.4%), did not receive permission from spouse/family (3.3%), transportation (1.9%) and HIV test cost too much (1.5%) (See Table 4).

**Table 4:** Reasons for not getting tested for HIV among adolescents and young adults 15–24 years of age in Tanzania (n = 4454, N = 5.2M, all numbers below in millions).

Reasons for not getting tested for HIV	N (%)
Don’t know	0.66 (11.83%)
Don’t need test/low risk	0.51 (9.15%)
Don’t know where to test	0.49 (8.74%)
Too far away	0.36 (6.4%)
Don’t want to know I have HIV	0.21 (3.74%)
Afraid others will know about test results	0.18 (3.17%)
Did not receive permission from spouse/family	0.17 (3.12%)
Transport costs too much	0.1 (1.77%)
Test costs too much	0.08 (1.4%)
Refused	0.07 (1.19%)
Test kits not available	0.04 (0.79%)
Afraid spouse/partner/family will know results	0.03 (0.57%)
Religious reasons	0.03 (0.53%)
Cannot get treatment for HIV	0.03 (0.47%)

### Factors associated with never testing for HIV among HIV-infected youth

Among those who were HIV-infected, confirmed by biomarker data, HIV-infected adolescents between 15–19 years of age were more likely to have never been tested for HIV than those 20–24 years of age (AOR = 4.5; 95% CI = 2.6–7.6; p<0.001). Those who were HIV positive and lived in a rural area were more likely to have never been tested for HIV than those who were HIV positive and living in urban areas (AOR = 4.3; 95% CI = 1.7–10.9; p<0.01). People living with HIV who had no education or lower than a primary school education were more likely to have never been tested for HIV than those with a secondary or post-secondary education (AOR = 17.7; 95% CI = 6.8–46.5; p<0.001 and AOR = 6.3; 95% CI = 3.1–12.7; p<0.001 respectively). High income groups living with HIV were more likely to have never been tested for HIV than low- and middle-income groups (AOR = 0.1; 95% CI = 0.0–0.3; p<0.01 and AOR = 0.1; 95% CI = 0.0–0.2; p<0.01 respectively). Single HIV-infected adolescents and young adults were more likely to have never been tested for HIV than those who were married (AOR = 4.1; 95% CI = 1.8–9.5; p<0.01). Finally, HIV-infected sex workers were more likely to have never been tested for HIV than HIV-infected individuals who did not buy or sell sex (AOR = 2.7; 95% CI = 1.4–5.4; p<0.01) (see Table 5).

**Table 5:** Adjusted odds ratio (AOR) for demographic and socio-behavioral characteristics associated with ever having tested for HIV among HIV-infected adolescents and young adults aged 15–24 in Tanzania (N = 0.14 million).

Variables	AOR (95% CI)
Gender	
Female	Reference
Male^‡^	0.37 (0.1, 1.46)
Age	
15–19^‡^	4.49 (2.64, 7.63)
20–24	Reference
Education	
No education^‡^	17.74 (6.77, 46.48)
Primary^‡^	6.26 (3.08, 12.72)
Secondary and above	Reference
Marital status	
Never married^‡^	4.09 (1.77, 9.47)
Married/living together	Reference
Separated/divorced	1.85 (0.89, 3.87)
Income	
Low	0.11 (0.03, 0.35)
Middle	0.05 (0.01, 0.23)
High	Reference
Residence	
Urban	Reference
Rural^‡^	4.35 (1.73, 10.91)
Age had sex for the first time	
Never had sex	Reference
< = 14 years^‡^	1.2 (0.27, 5.29)
>15^‡^	1.35 (0.37, 4.93)
Number of partners past 12 months	
0	Reference
1	0.71 (0.17, 2.96)
2+	0.94 (0.31, 2.86)
Use condoms during sexual intercourse	
No^‡^	1.33 (0.73, 2.41)
Yes	Reference
Buy or sell sex in past 12 months	
Yes	2.74 (1.38, 5.44)
No	Reference
Alcohol use	
Never drink	Reference
Occasional drink*	1.75 (0.9, 3.38)
Heavy drink	0.67 (0.15, 3.09)

* P<0.05

† P<0.01

‡ P<0.001

## Discussion

This study identified several demographic and socio-behavioral characteristics associated with HIV testing. In the weighted analysis, the proportion of HIV-infected youth aged 20–24 was almost three times of those aged 15–19. The proportion of females who were HIV positive was four times that of males. In the multivariate analysis, the adjusted odds of having ever been tested was higher among females (vs. males), those with a post-secondary education (vs. those with primary or no education), residents from urban settings (vs. rural settings), young adults between the ages of 20–24 (vs. those 15–19 years of age), those who are married or living with someone (vs. those who are not married) and those who reported using condoms during sexual intercourse (vs. those who do not use condoms during sexual intercourse). These findings are consistent with other studies conducted among Tanzania’s adolescent and young adult population [6, 10, 14–20].

Higher rates of HIV testing among female adolescents and young adults compared with males have been found in other studies [13]. It’s possible women have a ‘window of opportunity’ to get tested for HIV during prenatal or antenatal care [21]. WHO recommends universal HIV testing for all pregnant women and prompt treatment for HIV-positive women in HIV endemic countries to help prevent vertical transmission of the virus [22]. Among younger females, HIV testing was considered the best prevention strategy when they were unable to negotiate safer sex behaviors [23].

A possible explanation for the association of higher levels of education and HIV testing could be due to the fact that higher educational levels provide more opportunities to be educated about HIV infection and prevention which could then lead to more knowledge regarding where to access HIV counseling and testing resources, thus resulting in higher levels of HIV testing [12, 14, 17]. Higher testing in urban areas might be the result of urban areas offering greater access to HIV-related counseling and testing services than rural areas [12, 15]. The finding regarding 15–19 year olds being less likely to have ever tested than 20–24 year olds might be due to the fact that adolescent teens are less likely to know where to get tested than young adults [14, 17]. In the sample, most of adolescents 15–19 did not consider themselves at risk due to no sexual behaviors and therefore never had the HIV test. Structural issues such as travelling to testing centers, lack of confidential testing sites for adolescent teens and HIV-related stigma and discrimination might also explain this finding [9]. This suggest that mobile outreach services to young people might be beneficial in promoting and increasing HIV testing [24].

We found that adolescents and young adults who reported using condoms during sexual intercourse were more likely to get tested than those not using condoms. This finding is consistent with a previous study among men 15 years of age and older in Dar es Salaam, Tanzania which found that HIV testing was more common among men who consistently used a condom in the past 6 months [4]. However, it is important to note that 75% of our sample reported not using condoms when having sexual intercourse. Condom use continues to be one of the most effective methods for reducing the risk of HIV transmission [25]. Developing strategies on how best to promote the use of condoms in a culturally appropriate way for the young population in Tanzania is desperately needed. Introduction to use Pre-exposure prophylaxis (or PrEP) as prevention in the health infrastructure of Tanzania could be another strategy to lower HIV infection rates among youth [24].

To better inform the development of targeted HIV programs to reach the UNAIDS 95-95-95 goals, we predicted the factors that were associated with never having an HIV test among HIV-infected participants. HIV status was confirmed using biomarker data. HIV awareness included both perspectives that HIV-infected people were aware of their positive status and negative status. Our findings indicate programs focusing on HIV testing and education should target adolescents, those who are single, those with less than a primary school education, sex workers, high income groups and those living in rural areas. In resource-limited countries, intervention strategies may focus on improving the low-cost HIV self-test kits among youth [26].

Our study showed the majority of characteristics affecting HIV testing among young people in Tanzania have not changed over the years, thus it will be necessary to re-examine the current approaches to HIV prevention and testing. Innovative interventions should be implemented in Tanzania including PrEP, self-test, social media marketing, and community-wide testing service and management [27–29]. The COVID-19 pandemic will add to this challenge as it collides with the ongoing HIV epidemic and competes for needed medical supplies and health care provider resources, especially in resource-constrained areas such as sub-Saharan Africa where its affects will be compounded by high poverty rates and inadequately resourced health care systems [30–32]. There is increasing concern that COVID-19 will result in the disruption of HIV testing and treatment services leading to increased onward transmission and an excess of HIV-related deaths[30–33]. In light of this current situation, intensified and targeted HIV education and testing programs in high prevalence areas with the most at risk populations should be prioritized in Tanzania. WHO has recommended HIV self-testing as a strategy for increasing HIV testing rates during COVID-19. HIV self-testing affords individuals privacy, convenience, and empowering options for care [32, 34]. Other efforts to address some of the concerns regarding HIV testing in our sample (such as access to HIV testing, level of risk and fear of knowing HIV status) could include community-based mobilization for education and testing, voluntary and confidential counseling via telehealth options, more testing centers in rural areas and coming up with additional novel strategies for engaging the most affected populations in counseling and testing through culturally and linguistically appropriate social media platforms and other technology [1].

The study is a secondary analysis of PHIA conducted in Tanzania using the most recent data released in 2018 [4]. The selection of covariates, including demographic variables and socio-behavior variables were limited to information being collected by the THIS survey.

The findings in this paper using cross-sectional analysis could only be generalized to the adolescent and young adult population (aged 15–24) in Tanzania during the time of data collection. Given the current COVID-19 pandemic, the comparison between current available data with future data may show the impact of the pandemic on HIV testing among young people. This could inform future strategies to improving the testing rates among youth in Tanzania.

In conclusion, to meet the UNAIDS 95-95-95 fast-track targets and help end the AIDS epidemic among adolescents and young adults in Tanzania by 2030 [9], implementing targeted, high-impact behaviorally-focused HIV prevention and testing programs are crucial. Additionally, making confidential HIV testing services and treatment available and accessible to all regardless of age, gender, residential area (urban vs. rural) and income level will be key to reducing the onward transmission of HIV and HIV-related morbidity and mortality. Alternative testing approaches among young people should be implemented, such as including HIV self-testing rather than provider-initiated testing. Innovative strategies should be considered in Tanzania when promoting HIV testing among youth. The integration of testing and case management should be included in the health system.

## Supporting information

S1 Questionnaire PLOS’ questionnaire on inclusivity in global research. (DOCX)

S1 Data Analytical data for the study. (ZIP)
